# A Fundamental Role for Oxidants and Intracellular Calcium Signals in Alzheimer’s Pathogenesis—And How a Comprehensive Antioxidant Strategy May Aid Prevention of This Disorder

**DOI:** 10.3390/ijms22042140

**Published:** 2021-02-21

**Authors:** Mark F. McCarty, James J. DiNicolantonio, Aaron Lerner

**Affiliations:** 1 Catalytic Longevity Foundation, San Diego, CA 92109, USA; markfmccarty@gmail.com; 2 Saint Luke’s Mid America Heart Institute, Kansas City, MO 64111, USA; jjdinicol@gmail.com; 3 Chaim Sheba Medical Center, The Zabludowicz Research Center for Autoimmune Diseases, Tel Hashomer 5262000, Israel

**Keywords:** oxidants, anti-oxidants, oxidative stress, intracellular calcium, Alzheimer’s disease, prevention, amyloid β, phycocyanobilin, glutamate, glutathione synthesis

## Abstract

Oxidative stress and increased cytoplasmic calcium are key mediators of the detrimental effects on neuronal function and survival in Alzheimer’s disease (AD). Pathways whereby these perturbations arise, and then prevent dendritic spine formation, promote tau hyperphosphorylation, further amplify amyloid β generation, and induce neuronal apoptosis, are described. A comprehensive program of nutraceutical supplementation, comprised of the NADPH oxidase inhibitor phycocyanobilin, phase two inducers, the mitochondrial antioxidant astaxanthin, and the glutathione precursor N-acetylcysteine, may have important potential for antagonizing the toxic effects of amyloid β on neurons and thereby aiding prevention of AD. Moreover, nutraceutical antioxidant strategies may oppose the adverse impact of amyloid β oligomers on astrocyte clearance of glutamate, and on the ability of brain capillaries to export amyloid β monomers/oligomers from the brain. Antioxidants, docosahexaenoic acid (DHA), and vitamin D, have potential for suppressing microglial production of interleukin-1β, which potentiates the neurotoxicity of amyloid β. Epidemiology suggests that a health-promoting lifestyle, incorporating a prudent diet, regular vigorous exercise, and other feasible measures, can cut the high risk for AD among the elderly by up to 60%. Conceivably, complementing such lifestyle measures with long-term adherence to the sort of nutraceutical regimen outlined here may drive down risk for AD even further.

## 1. Introduction

### 1.1. Oxidant Stress and Intracellular Calcium Signals—Roles in Alzheimer’s Pathogenesis

**Figure 1:**
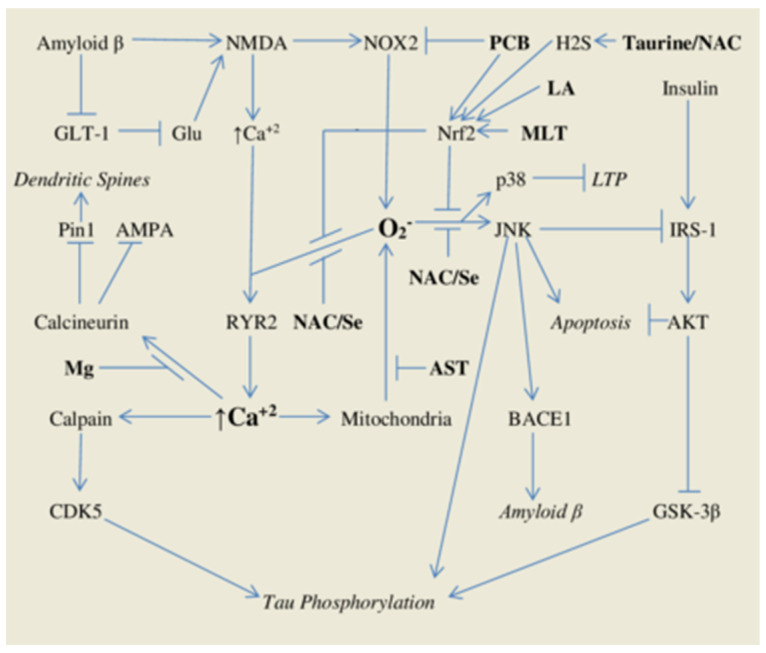
Intracellular calcium signals and oxidant production, triggered by amyloid β via N-methyl-D-aspartate (NMDA) receptors, collaborate to promote neuronal tau hyperphosphorylation, block formation of dendritic spines, promote amyloid β formation, suppress long term potentiation (LTP), and induce apoptosis. Antioxidant nutraceuticals with potential for intervening in this process are highlighted in bold.

Although the pathology of Alzheimer’s disease (AD) appears to be almost unimaginably complex, there seems to be fundamental agreement that elevated intracellular calcium levels and increased oxidant production play important roles in this regard. Figure 1 provides a depiction of how these factors may interact.

Whereas soluble amyloid β oligomers may address many cellular targets, extra- synaptic N-methyl-D-aspartate (NMDA) receptors may be of key importance in this regard. Via direct interaction and/or the ability of amyloid β oligomers to inhibit mechanisms of glutamate uptake by astrocytes and pre-synaptic membranes—resulting in extrasynaptic accumulation of glutamate—amyloid β activates extrasynaptic NMDA receptors, leading to an influx of calcium and a concurrent activation of NOX2-dependent NADPH oxidase complexes within their microenvironment [1,2,3]. These locally generated oxidants can oxidize ryanodine receptors (RYR2) in nearby endoplasmic reticulum (ER), such that they are far more likely to open in response to the modest calcium influx mediated by NMDA receptor activation [4]. This can be expected to lead to repeated “sparks” of calcium release from the ER, resulting in much more substantial local elevations of cytoplasmic calcium. Much of this released calcium will likely be “buffered” by uptake into nearby mitochondria. The resulting mitochondrial calcium overload will stimulate mitochondrial oxidative metabolism, leading to increased superoxide generation that complements the oxidant production stemming from NADPH oxidase; this phenomenon may also induce the mitochondrial fission that is typically seen in AD neurons [4].

The resultant oxidant stress can be expected to promote activation of the stress-activated MAP kinases, including c-Jun N-terminal kinase (JNK), known to be another key mediator of AD pathology [5], via increased activation of the upstream kinase apoptosis signal-regulating kinase (ASK1), or via induction of ER stress, which signals to JNK via IRE1alpha [6,7]. Persistent JNK activation may be largely responsible for the neuronal insulin resistance typical of AD, as JNK can confer an inhibitory phosphorylation on insulin receptor substrate-1 (IRS-1), a mediator of the insulin signaling pathway [8,9,10]. As a result, neuronal AKT activity is down-regulated; this is of importance both because AKT exerts anti-apoptotic activity via multiple effects, and also because AKT confers an inhibitory phosphorylation on glycogen synthase kinase-3β, one of the main kinases known to contribute to tau hyperphophorylation [8,11,12]. Moreover, JNK can phosphorylate tau directly, in a manner which prevents tau’s proteolytic cleavage [8]. Activation of the other stress-associated MAP kinase, p38, has been shown to mediate the suppression of neuronal long-term potentiation associated with amyloid β-mediated activation of extrasynaptic NMDA receptors; this presumably plays a key role in AD-associated cognitive dysfunction [2].

Increased cytoplasmic calcium can also activate calpain, a protease which, via conversion of the cdk5 activator p35 to p25, induces aberrant cdk5 activity that is a potent mediator of tau phosphorylation [13]. Furthermore, elevated calcium stimulates the activity of calcineurin, which acts in a number of ways to promote neuronal dysfunction in AD; in particular calcineurin impedes the formation of dendritic spines by inhibiting the Pin1 prolyl isomerase, and impairs synaptic function by inducing endocytosis of synaptic AMPA receptors [14,15].

While this survey of the pathogenesis of neural dysfunction and death associated with AD is indeed simplistic relative to the complex reality of this disorder, it does integrate a number of factors which appear to play an important role in this pathogenesis. The modest clinical utility of memantine in early AD is believed to stem from a selective partial inhibition of extrasynaptic NMDA receptors [16]. The ability of amyloid β to boost extracellular glutamate levels in the hippocampus may be largely attributable to suppressed membrane expression of the GLT-1 glutamate receptor in astrocytes; AD model mice with a concurrent heterozygous deficiency of GLT-1 develop accelerated cognitive dysfunction, whereas, in autopsy studies, patients with significant amyloid plaques tended to be cognitively normal at death when their astrocytes showed higher levels of GLT-1 expression [17,18,19]. Knockout of p47, an obligate component of the NOX2-dependent NADPH oxidase complex, has recently been shown to prevent cognitive dysfunction and tau hyperphosphorylation in two types of AD mouse models without significantly impacting soluble or insoluble amyloid β levels [19]. When mice are genetically modified to express modified forms of the RYR2 calcium channel that have reduced open times, cognitive function is likewise preserved in AD model mice; administration of the R-isoform of the drug carvedilol, which similarly down-regulates RYR2 open time, without blocking its opening, is similarly protective [20]. Genetically up-regulated expression of the mitochondrial Na-Ca exchanger preserves cognitive function in AD model mice, demonstrating a crucial role for mitochondrial calcium overload in AD [21]. Genetic or drug-mediated diminution of either calcineurin or JNK function aids cognitive preservation; as a clinical correlate, organ transplant recipients treated chronically with the calcineurin inhibitor FK506 have been found to be at notably lower risk for AD [5,22,23]. A partial reduction of tau expression is also protective in AD model mice.

### 1.2. Phycocyanobilin—A Phyconutrient NADPH Oxidase Inhibitor

Antioxidant nutraceuticals have the potential to intervene in these pathological processes in several ways. With respect to what appears to be the triggering source for oxidant stress in AD, NADPH oxidase, it is fortunate that nature has provided us with a practical tool for inhibiting such complexes. The unconjugated bilirubin generated by heme oxygenase activity has been found to be a highly potent physiological inhibitor of certain NADPH oxidase complexes, including NOX2-dependent activity; this doubtless reflects a homeostatic mechanism in which oxidative stress drives increased expression of heme oxygenase-1, and the resultant generation of bilirubin feeds back to inhibit a key source of oxidative stress [24,25,26]. Although bilirubin per se is too insoluble to be administered orally and its more soluble precursor biliverdin is quite expensive to synthesize, its antioxidant activity can be mimicked by a biliverdin-derived chromophore that constitutes about 0.6% of the dry weight of the traditional food spirulina [27]. Phycocyanobilin (PCB), a biliverdin metabolite which functions as a light-harvesting chromophore in cyanobacteria—such as spirulina—and certain blue-green algae, can mimic the ability of unconjugated bilirubin to inhibit NOX2-dependent NADPH oxidase; moreover, it is orally active in this regard when administered in intact spirulina or in spirulina extracts [27,28]. PCB can serve as a substrate for the ubiquitously expressed enzyme biliverdin reductase, responsible for converting biliverdin to bilirubin; this enzyme converts PCB to phycocyanorubin, a poorly soluble compound which is almost identical in secondary and tertiary structure to bilirubin; plausibly, it is this compound which is the direct mediator of PCB’s NADPH oxidase-inhibitory activity [29]. These considerations may explain, to at least some extent, the potent and versatile activity of orally administered spirulina (or of its primary protein, phycocyanin, to which PCB is covalently bound) in rodent models of a wide range of disorders in which oxidative stress is thought to play a pathogenic role [30,31,32,33,34]. Indeed, the bewildering range of disease models in which phycocyanin or spirulina has been found beneficial may reflect the fact that, in a wide range of pathologies, NADPH oxidase becomes activated in the affected tissues and either exacerbates or even mediates the syndrome [27]. Although the utility of PCB or of whole spirulina appears so far to have received little study in rodent AD models, its protective activity is well documented in rodent models of Parkinson’s disease and ischemic stroke—which demonstrates that it has access to the brain and can provide important antioxidant protection there [35,36,37,38,39,40,41,42].

### 1.3. Phase Two Induction and Support for Glutathione Synthesis

The up-regulatory effects of oxidants on both RYR2 and JNK activity are likely mediated by hydrogen peroxide, which exerts various pro-inflammatory effects via reversible oxidation of cysteine groups in susceptible proteins [43,44]. Such oxidations can be prevented by various peroxidase enzymes which catabolize hydrogen peroxide, and can be reversed by mechanisms dependent on the intracellular antioxidants’ glutathione and thioredoxin [45,46,47,48]. Activation of the Nrf2 transcription factor—so-called “phase two induction”—leads to increased synthesis of both glutathione and thioredoxin, as well as increased synthesis of peroxidases and of enzymes which maintain glutathione and thioredoxin in their active reduced forms [49]. Certain phytochemicals and metabolites known as phase two inducers—lipoic acid (LA) and sulforaphane (from broccoli sprouts) are often employed clinically in this regard—are susceptible to metabolic conversion to electrophiles, which can react covalently with cysteine groups in Keap1, a protein which binds to Nrf2 and retains it in the cytoplasm; this disrupts Keap1’s interaction with Nrf2, enabling the latter to migrate to the nucleus and promote transcription of its target antioxidant genes [50,51,52,53].

Other antioxidants boost Nrf2 activity, not by interaction with Keap1, but by promoting increased mRNA and protein expression of Nrf2. The neurohormone melatonin (MLT), secreted nocturnally by the pineal gland as a synchronizer of diurnal biological rhythms, is known to have this property [54]. This might reflect increased activity of the fundamental clock protein Bmal1, one of whose transcriptional targets is Nrf2 [55,56,57]. MLT administration has been found to benefit cognitive function, lessen tau phosphorylation, and decrease amyloid β levels in multiple models of AD in mice, albeit it is unclear to what extent antioxidant effects mediate these benefits [58,59,60,61,62]. Remarkably, PCB also has been reported to boost expression of Nrf2 and its transcriptional target heme oxygenase-1 (HO-1) [63,64]. Although the basis of this effect has not yet been clarified, it is notable that unconjugated bilirubin can function as an agonist for the aryl hydrocarbon receptor, one of whose functions is to promote transcription of the Nrf2 gene [65,66,67]; perhaps PCB shares this property of its chemical relative bilirubin. With respect to the key intracellular antioxidant glutathione, phase two induction boosts its level via induction of gamma-glutamylcysteine synthetase, the enzyme which is rate-limiting for glutathione synthesis [68]. However, the rate-limiting substrate for this synthesis is the amino acid cysteine, the tissue levels of which tend to decline during aging, leading to a reduction in glutathione levels [69]. The nutraceutical N-acetylcysteine (NAC) constitutes a safe and well-tolerated agent for boosting plasma and tissue levels of cysteine—and hence is employed for boosting tissue glutathione levels [70,71]. In mice given intrahippocampal injections of amyloid β oligomers, dietary pretreatment with NAC was found to normalize RYR2 expression and function, as well as cognitive function [72].

NAC may also provide protection by serving as a precursor for synthesis of hydrogen sulfide (H_2_S) [73]. Boosting brain levels of this endogenous gaso-transmitter, via intraperitoneal of sodium hydrosulfide, has been found to ameliorate pathology and lessen cognitive decline in multiple mouse AD models [74,75,76,77,78,79,80]. As H_2_S is known to promote Nrf2 activation, that might explain this protection in part, but other effects are likely to be involved [76,80]. There is also reason to suspect that the antioxidant taurine may promote brain synthesis of H_2_S [81]. The vascular protective properties of this agent have recently been traced to the ability of this compound to induce H_2_S-generating enzymes in vascular tissue [82]. The chief source of H_2_S in the brain is the enzyme cystathionine beta-synthase (CBS), and taurine has recently been reported to sustain CBS expression in the face of hemorrhagic stroke in rats; hence, it has been proposed that some of the cerebro-protective effects of taurine reflect enhanced or maintained CBS expression and a consequent increase in brain H_2_S levels [81,83]. Consistent with these speculations, the utility of dietary taurine has been reported in AD model mice; curiously, taurine binds directly to amyloid β oligomers [84,85]. CBS is allosterically activated by S-adenosylmethionine, and epidemiology correlating increased dietary intake of nutrients which promote methyl group availability (e.g., folate, vitamin B12, betaine) to lower AD risk might possibly be explained by enhanced brain CBS activity [81].

The micronutrient selenium (Se) is an obligate cofactor for certain phase two-inducible antioxidant enzymes—notably the various isoforms of glutathione peroxidase and thioredoxin reductase; the activity of these enzymes will therefore be diminished in people whose Se intake is below some minimal level [86]. The Se status of crops tends to vary directly with soil Se level, as Se is not nutritionally essential for plants [87]. The inconsistent results of epidemiology correlating plasma Se with health outcomes, and of Se supplementation trials, may reflect the fact that supplemental Se only boosts the activity of Se-dependent enzymes in subjects whose baseline Se status is relatively low. Consistent with this, trials of Se supplementation find that it lowers cancer risk and aids cardiovascular health only in subjects with relatively low plasma Se at baseline [88,89]. A meta-analysis of Se cancer chemoprevention trials found Se to be protective in low-Se regions, but not elsewhere [90]. Hence, it is reasonable to suspect that, at least in areas where soil Se can be low, people consume locally produced foods, and suboptimal Se status consequently is common, Se intake will correlate inversely with risk for AD or cognitive decline. Some epidemiology from regions where soil Se is frequently low supports this view [91,92,93,94]. People with plasma Se below 100 ng/mL and intakes below 70 mcg daily are most likely to benefit from supplemental Se. In low-Se regions, vegans tend to be at higher risk for low Se intakes, since animals store Se in selenoenzymes, but plants do not [95,96].

## 2. A Comprehensive Antioxidant Strategy May Aid Prevention of Alzheimer’s Disease

### 2.1. Astaxanthin—Antioxidant Protection for Calcium-Overloaded Mitochondria

With respect to oxidant production by calcium-overloaded mitochondria, it appears likely that the carotenoid astaxanthin (AST) has potential protective utility in this regard. AST is precisely configured to provide scavenging antioxidant protection to bilipid membranes, such as the inner mitochondrial membrane, which is notably subject to oxidative damage owing to its high content of unsaturated fatty acids and its capacity to generate superoxide via “electron slippage”; hence astaxanthin is thought to have outstanding potential as a mitochondrial antioxidant and as a “brain food” [97,98,99]. In calcium-overloaded mitochondria, Krebs cycle activity is boosted owing to calcium’s allosteric impact on several Krebs cycle enzymes [100]. This forces more electrons down the electron transport chain (ETC), increasing the rate of superoxide generation. It is reasonable to suspect that, under these circumstances, oxidative damage to the ETC will reduce the efficiency of electron transport, further increasing the propensity for electron slippage and superoxide generation; hence, by preventing such damage, AST may help to minimize oxidant production by calcium-overloaded mitochondria. Notably, oral astaxanthin has been found to confer cognitive protection in APP/PS1 double transgenic mice [101,102].

### 2.2. Controlling Amyloid β Production via Modulation of BACE1 and ADAM10 Expression

**Figure 2:**
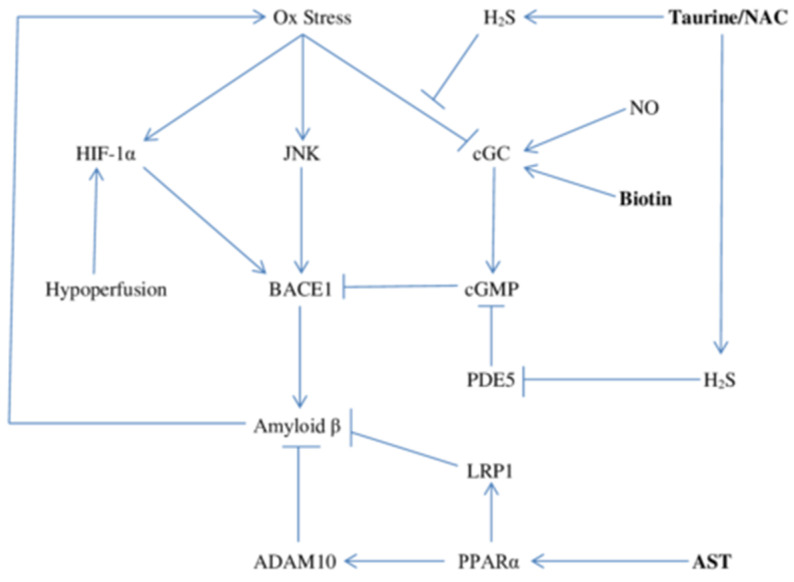
Modulation of BACE1 and ADAM10 expression and consequent amyloid β production by oxidative stress, hypoperfusion, determinants of cGMP production, and PPARα agonism. Nutraceutical measures which support or mimic NO bioactivity (biotin), promote H_2_S biosynthesis (taurine/NAC), and activate PPARα (AST) may down-regulate amyloid β synthesis.

The protease BACE1 (a.k.a. beta-secretase) cleaves the membrane-associated amyloid precursor protein (APP) in a way that destines it to be further cleaved via gamma-secretase to generate amyloid β monomers; it is a functional competitor of ADAM10 (alpha-secretase), which routes APP to non-pathogenic peptides [103]. Hence, modulation of BACE1 and of ADAM10 expression is a key determinant of the rate at which amyloid β is generated. Figure 2 summarizes some relationships which influence BACE1 and ADAM10 expression.

Brain hypoperfusion, and consequent intermittent or chronic hypoxia, can activate the hypoxia-inducible factor-1alpha (HIF-1α) by opposing its hydroxylation by a prolyl isomerase, and thereby preventing its ubiquitination and proteasomal degradation [104,105]. Oxidative stress likewise up-regulates HIF-1α levels by suppressing its prolyl hydroxylation [106,107]. In conjunction with its binding partner ARNT (a.k.a. HIF-1β), HIF-1α acts as a transcription factor, promoting the expression of certain genes that enable adaptation to hypoxia [108]. In this regard, HIF-1α has been found to bind to the promoter of the gene encoding BACE1, promoting its transcription [109,110]. This phenomenon likely helps to explain the well-known association between cerebrovascular disease and stroke and AD risk [110]. The MAP kinase JNK, which as we have seen, is also activated by oxidative stress, also up-regulates BACE1 expression at the transcriptional level; the basis for this effect remains unknown [111,112]. On the other hand, enhanced levels of cGMP suppress BACE1 expression [113,114]. It has been speculated that this effect is attributable to a cGMP-mediated increase in PGC-1α (PPARδ coactivator-1α) [113,115,116]. PGC-1α is a coactivator for certain transcription factors including PPARδ, which is known to bind to the promoter of the BACE1 gene [117,118]. Overexpression of PGC-1α inhibits BACE1 expression at the transcriptional level, and this effect is blocked by concurrent PPARδ knockout [118].

Antioxidant nutraceutical strategies therefore have potential for decreasing BACE1 by blunting activation of both HIF-1α and JNK. Oxidative stress also can oppose cGMP production via oxidation of the soluble guanylate cyclase (sGC); such oxidation converts the heme iron in this enzyme to ferric state, preventing nitric oxide (NO) from generating cGMP in response to NO, and also rendering sGC more prone to proteasomal degradation [119]. Fortunately, oxidized cGC is temporarily susceptible to restorative reduction by H_2_S, which reduces the ferric iron to ferrous form (+2); indeed, one of H_2_S’s key physiological roles may be to maintain cGC activity in the face of oxidative stress [120]. Furthermore, H_2_S can boost cGMP levels via direct inhibition of phosphodiesterase 5 [121,122]. As we have seen, supplementation with taurine and NAC has potential for boosting brain generation of H_2_S.

A further way in which brain cGC can be activated—at least while it is in its effective reduced form—is via interaction with supraphysiological levels of the B vitamin biotin [123,124]. In concentrations roughly two orders of magnitude higher than its physiological plasma level, biotin serves as effective mimic of NO or carbon monoxide as an activator of cGC; this rationalizes certain protective effects of high-dose biotin in rodent models of diabetes and hypertension, and possibly helps to explain the recently demonstrated utility of high-dose biotin in the management of multiple sclerosis [124,125,126,127]. High-dose biotin tends to be well tolerated because it is capable of boosting cGC activity by only two- to three-fold, as opposed to the much larger activation that can be achieved with elevated levels of NO, which can precipitate dangerous hypotension [123]. Although daily biotin intakes of up to 300 mg have been employed in MS treatment, it seems likely that intakes about an order of magnitude less would be capable to achieving a physiologically meaningful increase in cGMP production, with minimal risk for side effects. The chief drawback to such supplementation is that biotin can interfere with certain clinical assays that employ biotinylated reagents; hence, discontinuing high-dose biotin prior to conducting such assays is advisable [128]. The impact of high-dose biotin in mouse AD models has not yet been studied, but this could readily be accomplished.

As noted, ADAM10 cleaves APP in such a way as to preclude its later conversion to amyloid β by BACE1. Hence, measures which safely up-regulate ADAM10 expression oppose amyloid b generation. The transcription factor peroxisome proliferator-activated receptor α (PPARα) binds to the promoter of the PPARα gene, and can drive its transcription [129]. Remarkably, AST can act as a PPARα agonist in physiologically relevant low nanomolar concentrations [130,131]. At 10 nM, AST was found to double the mRNA expression of ADAM10 in primary porcine brain capillary endothelial cells [132]. Moreover, AST, presumably via PPARα agonism, also increased endothelial expression of lipoprotein receptor-related protein 1 (LRP1), a protein expressed on the abluminal surface of brain capillaries that functions to transport amyloid β monomers and oligomers from the brain [132,133]. Importantly, genetic knockout of LRP1 in 5XFAD AD model mice elevates their brain levels of soluble amyloid β and exacerbates their cognitive dysfunction [133]. Hence, AST has the potential to decrease AD risk both by serving as a potent mitochondrial antioxidant, and by acting as a PPARα agonist that opposes amyloid β generation and promotes its export from the brain.

In summary, nutraceutical measures which mimic the biological activity of NO by boosting cGMP, that promote H_2_S biosynthesis, or that boost PPARα activity, may complement antioxidant measures in suppressing the production of amyloid β.

### 2.3. Antioxidants May Support Astrocyte Glutamate Uptake

As noted, the activation of extrasynaptic NMDA receptors that appear to be the key drivers of amyloid β neurotoxicity is mediated, in whole or in part, by a failure of astrocytes and pre-synaptic membranes to clear glutamate efficiently from synaptic clefts, leading to glutamate spillover. Amyloid β-mediated down-regulation of the membrane expression of the glutamate transporter glutamate transporter-1 (GLT-1, a.k.a. EAAT1) is thought to be primarily responsible for this; this effect is post-translational, as the mRNA for GLT-1 is not altered [18,134]. Amyloid β, via interaction with beta1-integrin, provokes protein kinase C (PKC) activation in astrocytes, and treatment of astrocytes with PKC-activating phorbol esters have been shown to enhance ubiquitination of GLT-1 by the E3 ubiquitin ligase Nedd4-2, leading to clathrin-dependent endocytosis of GLT-1 that can promote its lysosomal degradation [135,136,137,138,139].

Although the signaling pathway whereby amyloid β provokes GLT-1 ubiquitination and endocytosis requires further clarification, there is reason to suspect that activation of Nox2-dependent NADPH oxidase, and consequent activation of JNK, may be responsible. Amyloid β is known to induce the astrogliosis and glial fibrillary acidic protein (GFAP) overexpression typical of AD by an interaction with beta1-integrin which leads to PKC-mediated activation of Nox2 NADPH oxidase; inhibitors of NADPH oxidase prevent this astrogliosis and GFAP induction [135]. JNK is also activated by amyloid β in astrocytes; JNK promotes GFAP expression via c-Jun, and its inhibition likewise blocks GFAP induction and astrogliosis [140,141]. Three lines of evidence suggest that JNK activation may be responsible, at least in part, for GLT-1 endocytosis in amyloid β-treated astrocytes. JNK has been shown to confer a phosphorylation on Nedd4-2, at Thr899 in its catalytic domain, that enables it to ubiquitinate and promote endocytosis of the endothelial sodium channel ENaC; it is reasonable to suspect that such phosphorylation likewise enables Nedd4-2 to ubiquitinate GLT-1 [142,143]. Secondly, insulin opposes the ability of amyloid β to down-regulate GLT-1; as we have seen, JNK can down-regulate insulin signaling via phosphorylation of IRS-1 [144]. Thirdly, PKC, an activator of NADPH oxidases, is known to provoke GLT-1 ubiquitination and endocytosis. Hence, a reasonable hypothesis, susceptible to experimental verification, is that amyloid β promotes GLT-1 endocytosis in astrocytes via activation of NADPH oxidase and its downstream target JNK. If this view is correct, then most likely the antioxidants recommended here for down-regulating NADPH oxidase-JNK signaling in neurons would provide similar benefit in amyloid β-afflicted astrocytes, and hence support the ability of astrocytes to clear glutamate efficiently.

### 2.4. Antioxidants May Sustain Activity of Amyloid β-Degrading Proteases

Although brain and CSF levels of BACE1 have been found to be elevated in AD patients and to correlate with brain amyloid load and decrease in hippocampus volume, and though increased generation of amyloid β is clearly the driving pathogenic factor in early-onset AD patients carrying certain variant genes, a classic study comparing brain turnover of amyloid β in patients with sporadic AD and healthy controls concluded that a diminished capacity to catabolize or export amyloid β is the chief reason why amyloid β accumulates in the brain of sporadic AD patients [145,146,147,148,149,150]. Extracellular and intracellular proteases are capable of degrading amyloid β monomers and oligomers, microglia can phagocytize fibrillar amyloid β, amyloid β monomers/oligomers can be endocytosed by a range of membrane receptors expressed by glial cells and neurons, amyloid β can be cleared through the “glymphatic” system, and blood-brain barrier capillaries are capable of endocytosing amyloid β monomers/oligomers abluminally and expelling them into the bloodstream [151,152,153,154,155]. Up-regulating any of these mechanisms has the potential to aid in the prevention of AD.

Several proteases expressed by the brain can act extracellularly to degrade amyloid β monomers and oligomers, most notably neprilysin and insulin-degrading enzyme (IDE) [151]. Neprilysin is a type 2 membrane metalloendopeptidase whose active site is in its extracellular carboxy-terminal domain; unlike IDE it can degrade both monomers and oligomers of amyloid β42 [156]. Neprilysin expression tends to be decreased in the AD-afflicted portions of the brain of AD patients [157,158,159]. This seems likely to be of pathogenic significance, since, in rodent AD models, knockout or inhibition of neprilysin exacerbates the typical pathology and cognitive dysfunction, whereas measures which enhance neprilysin expression have an ameliorative impact in that regard [160,161,162]. Also of note is the fact that neprilysin specific activity tends to be decreased in AD patients and rodent or cellular models, suggestive of post-translational inhibitory modification [163,164].

Some researchers have demonstrated that oxidative stress can inhibit neprilyin activity without influencing its expression. In particular, they have shown that a highly reactive product of polyunsaturated fatty acid oxidation, 4-hydroxynonenal, can covalently bind to and inactivate neprilysin in oxidatively stressed cells [165,166,167]. In a recent study of particular interest, a neuroblastoma-derived cell line was exposed to amyloid β oligomers for 12 h, with or without concurrent incubation with NAC or the phytochemical scavenging antioxidant xanthorrhizol [168]. Whereas incubation with amyloid β alone caused neprilysin activity to fall by over 50%, co-incubation with either NAC or xanthorrhizol was shown to reverse this reduction dose-dependently. A very similar effect was seen when the cells were exposed to 4-hydroxynonenal rather than amyloid β.

Perhaps surprisingly, it is hard to find studies that have examined the impact of antioxidant measures on brain neprilysin activity in rodent AD models. Nonetheless, these considerations suggest that a broad-spectrum antioxidant strategy of the type advocated here might well help to conserve neprilysin activity in such models; this could be tested readily. As a very potent membrane antioxidant, astaxanthin might have particular utility for suppressing the membrane lipid peroxidation which gives rise to 4-hydroxynonenal, the putative mediator of the adverse impact of oxidative stress on neprilysin activity [169,170].

Additional lifestyle and nutraceutical measures may have potential for elevating brain neprilysin expression and activity. Exercise training has been shown to have this effect in mice, whereas brain hypoperfusion or hypoxia tends to reduce neprilysin expression—an effect which might mediate in part the increased risk for AD that accompanies cerebrovascular disease [171,172,173]. Other reports indicate that oral administration of the green tea catechin epigallocatechin-3-gallate (EGCG) or prebiotic fructo-oligosaccharides can increase brain expression of neprilysin in AD model mice, whereas vitamin D deficiency can decrease brain neprilysin expression in mice [174,175,176]. These intriguing leads merit follow up.

### 2.5. Could Antioxidants Aid Expulsion of Amyloid β from the Brain?

Amyloid β oligomers act on brain capillary cells to suppress expression of both LRP1 and of P-glycoprotein (P-GP); LRP1 acts in the abluminal capillary membrane to extract amyloid β monomers/oligomers from the brain extracellular space, and P-G acts on the luminal side to expel them into the circulation [177,178,179,180,181,182,183]. Hence, they act in tandem to expel intact amyloid beta from the brain. As might be expected, LRP1 and P-GP are decreased in the brains of AD patients and of AD model mice, and there is reason to suspect that this has a functionally important impact on the brain’s amyloid β load [133,177,178,184,185]. Amyloid β also stimulates Nox2-dependent NADPH oxidase activity in brain endothelial cells via the receptors CD36 and RAGE—a phenomenon responsible for loss of arteriolar adaptive vasodilation in cerebral amyloid angiopathy [186,187,188,189]. The extent to which oxidant signaling mediates amyloid β-induced down-regulation of the LRP1 and P-glycoprotein in brain capillaries merits investigation.

Oxidant stress can increase expression of the sterol regulatory element-binding protein 2 (SREBP-2) transcription factor and its precursor protein in endothelial cells; moreover, SREBP-2 can bind to the promoter of the LRP1 gene, suppressing its transcription [190,191,192,193]. Hence, amyloid β-mediated activation of NADPH oxidase might suppress LRP1 expression by inducing SREBP-2 activity. In regard to P-GP, Aβ40 has recently been shown to provoke its ubiquitination and proteasomal degradation in endothelial cells [182]. This is likely to reflect increased expression of the ubiquitin ligase NEDD4-1; this enzyme is elevated in the brain capillaries of TG2576 AD model mice, and is known to utilize P-GP as a substrate [194]. The FOXM1B transcription factor promotes transcription of the NEDD4-1 gene, and is itself inducible by oxidant stress [195,196]. Indeed, FOXM1 serves as a detector of oxidant stress, providing homeostatic control by boosting expression of certain antioxidant enzymes [196,197]. How oxidant stress induces FOXM1 is not clear, though hypoxia-inducible factor might play a role in this regard [198,199]. Other cell culture data points to receptor for advanced glycation end products (RAGE)-mediated activation of NF-kappa B (known to be NADPH oxidase dependent [200]) as a factor in Aβ-mediated down-regulation of P-GP expression in brain endothelium [181]. These considerations suggest the desirability of examining the impact of PCB and other NADPH oxidase inhibitors on amyloid β-mediated down-regulation of LRP1 and P-GP expression in brain capillaries.

Of related interest is the fact that fish oil feeding has been shown to support brain expression of LRP1 and enhance amyloid β export from the brain in AD model mice [201]. This might reflect inhibition of SREBP-2 expression, as fish oil has been shown to have this effect in other cellular contexts [202,203]. These findings might help to rationalize epidemiology correlating increased intakes of fish and the omega-3 fatty acid docosahexaenoic acid (DHA) with reduced risk for dementia and specifically AD [204,205]. Remarkably, a meta-analysis of 21 prospective cohort studies calculated that a 100 mg/day increment in DHA consumption was associated with a 37% reduction in AD risk (RR 0.63, 95% CI: 0.51–0.76, *p* < 0.001) [205]. Curiously, brain uptake of DHA is inefficient in carriers of ApoE4, prone to earlier onset of AD [206]. 

### 2.6. Antioxidants May Support Cerebrovascular Endothelial Nitric Oxide Synthase Activity

A further potential consequence of amyloid β-induced oxidative stress in cerebral endothelial cells is the uncoupling of the endothelial nitric oxide synthase NOS (eNOS), as demonstrated in AD model mice [207]. The peroxynitrite that arises from the spontaneous reaction of superoxide with NO can oxidize tetrahydrobiopterin, essential cofactor for all isoforms of nitric oxide synthase (NOS), such that NOS becomes uncoupled, producing superoxide rather than nitric oxide [208]. Oxidative stress can also promote NOS uncoupling by inhibiting dimethylarginine dimethylaminohydrolase (DDAH), the enzyme which catabolizes asymmetric dimethylarginine (ADMA); ADMA, that arises by catabolism of proteins in which arginine has been post-translationally methylated, uncouples NOS by competing with its substrate arginine for binding to NOS [209]. It is reasonable to expect that neuronal NOS will likewise be uncoupled to some degree in neurons subjected to oxidative stress, as in AD—though this issue has received little study.

Effective eNOS activity is a mediator of homeostatically appropriate flow-induced vasodilation of cerebral arterioles [210]. In glutamate-responsive neurons, the calcium influx triggered by glutamate release boosts neuronal NOS activity, resulting in vasodilation of adjacent cerebral arterioles [211]. In these ways, effective NOS activity helps to boost blood flow to regions of the brain that are metabolically active. This adaptive mechanism may be impaired owing to oxidant-mediated uncoupling of NOS in AD.

As noted above, NO-induced cGMP can decrease BACE1 expression, thereby down-regulating amyloid β synthesis. In addition, the NO produced by eNOS—and presumably also nNOS—works to counteract excessive activation of calpain in brain neurons, possibly via direct S-nitrosylation of this protease [212,213,214]. In AD transgenic model mice, concurrent knockout of eNOS up-regulates calpain activity, p25 generation, CDK5 activity, and consequent tau phosphorylation [215]. Conversely, injections of the NO donor S-nitrosoglutathione have been shown to decrease the elevated tau phosphorylation in the brains of rats subjected to bilateral common carotid artery occlusion [216].

It is reasonable to suspect that supplemental PCB, by inhibiting amyloid β-triggered, NADPH oxidase-mediated endothelial or neuronal superoxide generation, may prevent oxidation of tetrahydrobiopterin and support effective NOS activity, thereby opposing hyperphosphorylation of tau. PCB and complementary antioxidants might also be expected to support DDAH activity, and thereby alleviate the eNOS uncoupling activity of ADMA.

The uncoupling activity of ADMA can also be counteracted by supplemental citrulline, which, by efficient enhancement of tissue arginine levels, boosts the cellular ratio of arginine to ADMA and thereby promotes coupled NOS activity [217,218,219]. In this regard, plasma levels of ADMA tend to be elevated in AD patients, suggesting that supplemental citrulline might aid the proper function of the cerebral vasculature and systemic vasculature of AD patients [220,221]. The impact of citrulline in AD model mice has yet to be reported; however, it is notable that supplemental citrulline has been reported to favorably impact the diminished LTP of aging rats [222]. Conceivably, this effect may reflect better coupling of neuronal NOS, which is a mediator of LTP [223,224].

## 3. Potential Enhancers of Amyloid β Neurotoxicity

### 3.1. Microglial Production of Interleukin-1β Potentiates Amyloid β Neurotoxicity

The neurotoxicity of amyloid β appears to be mediated in part by promotion of pro-inflammatory M1 differentiation of microglia, which leads to inflammasome activation and consequent production of interleukin-1β (IL-1β). Amyloid β achieves this via interaction with microglial toll-like receptors 2 and 4 (TLR2/TLR4) [225,226,227,228,229]. Stimulation of microglial TLR4 results in NLRP3-dependent inflammasome activation; the resulting IL-1β production has been found to exacerbate neuronal and cognitive dysfunction in rodent AD models, at least in part by amplifying tau phosphorylation [230,231,232,233,234,235,236]. Acting via neuronal IL-1 receptors, IL-1β can activate the MAP kinases JNK and p38, which as we have seen are also mediators of the neurotoxic effects of amyloid β [237,238]. Moreover, IL-1β has also been found to enhance the calcium influx via NMDA receptors provoked by glutamate, an effect dependent on p38 MAP kinase activation; the mechanistic basis of this effect still requires clarification [237,238,239,240,241,242]. Therefore, the IL-1β production associated with M1 microglial differentiation may act on neurons at a fundamental level to up-regulate the neurotoxic effects of amyloid β. On the other hand, M2-differentiated microglia can exert a protective effect in AD, as they have increased capacity to catabolize amyloid β via endocytic or phagocytic uptake [152,243,244].

A recent discussion of microglial activation following brain trauma—which likewise is mediated by activation of TLR2/LTR4, in this case by the damage-associated molecular pattern protein high-mobility group box protein 1 (HMGB1) released by dead or injured neurons [245]—has proposed that, in addition to the range of antioxidant nutraceuticals cited in this essay, vitamin D, long-chain omega-3 fatty acids, glucosamine, the green tea catechin EGCG, the soy isoflavone genistein, and the anti-diabetic phytochemical berberine may have practical potential for opposing pro-inflammatory M1 activation of microglia while promoting M2 differentiation [246]. The interested reader is referred to that article for the evidence supporting these conclusions. Hence, these nutraceuticals might be expected to aid prevention or control of AD by inhibiting microglial IL-1β production while promoting a neuroprotective M2 microglial phenotype. Indeed, favorable effects of vitamin D, DHA, EGCG, and berberine have been reported in mouse AD models [174,247,248,249,250,251,252,253,254,255,256,257,258]. Fortunately, these agents appear likely to promote health in a number of additional ways. The long chain omega-3 fatty acid DHA is of particular interest, since it can both dampen microglial inflammation and promote export of amyloid β from the brain. As noted previously, prospective epidemiology correlates modest increases in dietary DHA intakes with a large decrease in AD risk [205]. Recent meta-analyses of prospective cohort studies likewise find that plasma levels of 25-hydroxyvitamin D correlate inversely with AD risk [259,260,261,262].

### 3.2. Magnesium Deficiency May Up-Regulate Amyloid β Neurotoxicity

Since increased cytosolic free calcium is a key mediator of the toxic impact of amyloid beta oligomers on neurons, and magnesium has been described as “nature’s calcium blocker” [263], it is natural to wonder whether enhancement of intra-neuronal magnesium (Mg) through supplementation or a Mg-rich diet might have a down-regulatory impact on the some of the calcium-driven mechanisms that impair neuronal function in AD. In this regard, the binding of Ca to calmodulin—required for calcineurin activation and several other mechanisms whereby Ca dysregulation impedes neuronal function [264]—is opposed by Mg as it rises into the high-physiological intracellular range [265,266]. Hence, moderate intracellular Mg deficiency could be expected to up-regulate Ca activation of calmodulin.

Brain and cerebrospinal Mg levels are reported to be low relative to controls in AD patients. Prospective epidemiological studies have found that risk for dementia and for mild cognitive impairment AD is lower in people with relatively high dietary magnesium [267,268]. Furthermore, in subjects assessed for use of Mg oxide as a laxative, risk for developing dementia over a decade of follow-up was significantly lower than for those not taking this compound; after adjustment for various potential confounders, the adjusted hazard ratio was 0.517 (95% CI 0.45–0.79, *p* = 0.001) [269]. In a transgenic mouse model of AD, supplementation with Mg L-threonine—shown to boost cerebrospinal fluid Mg levels in mice more effectively than Mg gluconate, presumably owing to greater blood-brain barrier transport—was found to suppress amyloid β generation, preserve synapses, and aid maintenance of cognitive function [270]. This benefit of Mg was not observed when calcineurin was concurrently inhibited, suggesting that suppression of calcineurin activation may be largely responsible for Mg L-threonine’s benefit in this model. 

Good Mg status may also aid control of inflammation—dietary Mg intake correlates inversely with serum C-reactive protein, and supplemental Mg has been found to decrease this parameter [271,272]. Possibly, this reflects the fact that calcium/calmodulin-dependent kinase-II activation exerts an up-regulatory effect on NF-kappa B activity in macrophages, an effect also seen in microglia [273,274]. These considerations suggest that ensuring good Mg status with a whole-food diet and/or supplementation may provide a measure of protection from AD. And the impact of Mg L-threonine per se clearly merits further study, in light of its ability to elevate extracellular Mg in the brain of normally-fed mice. In this regard, a recent double-blind 12-week study with supplemental Mg L-threonine has found it to improve cognitive ability and remediate executive function deficits in cognitively impaired older adults, and an open-label 8-week study with this supplement reported significantly increased MMSE scores in patients with mild to moderate dementia [275,276].

## 4. Toward an Integrated Nutraceutical/Lifestyle Strategy for Amyloid β Neurotoxicity in Alzheimer’s Disease

Taken together, these considerations suggest that a nutraceutical regimen incorporating physiologically meaningful doses of PCB (as whole spirulina or PCB-rich spirulina extracts), a clinically effective phase two inducer such as LA, as well as other nutraceutical antioxidants such as melatonin, NAC, taurine, Se, and AST, may work in a cooperative fashion to minimize the multifarious contributions of elevated oxidant production to the pathogenesis of AD, while also evoking the neuroprotective effects of H_2_S. Including high-dose biotin in such a regimen may provide a complementary benefit by enhancing brain production of cGMP, which opposes BACE1 expression and consequently amyloid β generation. Adding additional components, such as vitamin D, DHA, EGCG, and berberine, may lessen the contribution of IL-1β to AD pathogenesis, while aiding microglial clearance of amyloid β. The utility of such a program is readily susceptible to assessment in rodent models of AD entailing elevated amyloid β production, and, if pre-clinical studies confirm important efficacy, would lend itself to practical implementation as a clinical strategy for preventing and, perhaps, slowing the progression of AD and indeed a range of other disorders in which oxidant stress plays an important pathogenic role.

There is growing evidence that certain prudent lifestyle measures have important potential for AD prevention. Recent meta-analyses of prospective epidemiology strongly suggest that healthy dietary patterns rich in plant-based whole foods, low in saturated fat-rich animal products, and incorporating fish omega-3s, as well as regular vigorous physical exercise, may markedly lower risk for AD; after multivariable adjustments, odds ratios were calculated of 0.61 (95% CI: 0.47, 0.79) and 0.62 (95% CI 0.49, 0.75), respectively [277,278,279]. Regular sauna use has also emerged recently as a likely protective lifestyle factor [280]. Not smoking, moderate consumption of ethanol, and cognitive exercise in late life have been found to be associated with lower AD risk. One intriguing recent study defined five lifestyle determinants of AD risk—not smoking, moderate alcohol consumption, a prudent Mediterranean or plant-based diet, regular vigorous physical exercise, and late-life cognitive exercise—and, employing data from two large longitudinal studies targeting an aging population, found that risk for incident AD risk was 60% lower in people who practiced four or five of these lifestyle activities, as opposed to zero or one (OR 0.40, 95% CI 0.28–0.56) [281].

Improved cerebrovascular health, diminished microglial inflammation, heat shock protein induction, increased production of neurotropic factors systemically and in the brain, and episodic exposure to lactic acid have been postulated as possible mediators of the protection from AD afforded by regular vigorous exercise [282,283,284,285,286,287]. Elevated levels of the “longevity hormone” fibroblast growth factor 21 (FGF21) and of adiponectin might help to explain the lower risk for AD observed in long-term vegans and quasi-vegan societies, as these hormones are protective in mouse AD models [288,289,290,291,292,293,294]. Strikingly, the Ibadan-Indianapolis Dementia Project, a longitudinal study following elderly African-Americans in Indianapolis and elderly Africans in Ibadan during the 1990s, and using standardized diagnostic criteria, found that age-adjusted incidence of AD was less than half as high in the latter group [295]. The diet of the Yoruba residents of Ibadan at the time was said to consist of grains, roots, tubers, and a small amount of fish [296]. Cyclic restriction of dietary essential amino acids—mimicking the relatively low intakes of certain essential amino acids associated with many vegan diets—reduces cognitive decline and tau phosphorylation in the triple transgenic AD mouse model [297]. However, low consumption of saturated fat likely contributes to the protection afforded by quasi-vegan as well as by “prudent” omnivore diets [298]. A diet rich in saturated fatty acids up-regulates pro-inflammatory activation of microglia in rodents, possibly accounting for the adverse impact of saturated fat-rich diets on AD risk [299,300]. Palmitic or stearic acid can activate microglia in vitro through a mechanism dependent on TLR4 [301]. And elevated low-density lipoprotein cholesterol (>121 mg/dL)—less common in those following vegan or Mediterranean diets—is associated with AD risk; whether this association is causative remains unclear [302,303,304]. (As a cautionary note—vegans would be well advised to supplement with DHA, Se, and of course vitamin B12). Anti-inflammatory effects of the ethanol metabolite acetate might mediate the protection from AD afforded by light/moderate alcohol consumption [305,306,307]. It is certainly highly encouraging that AD appears to be a disease that is quite susceptible to prevention by a prudent lifestyle.

However, another and less optimistic way to look at this is that, even aging people who have make every reasonable effort to follow a prudent lifestyle are still confronted with 40% of the high risk for a devastating late-life decline in cognitive function that is faced by those who make little if any effort to promote their own health. This is why it would be a wonderful development if health-oriented people had a regimen of nutraceuticals and perhaps very safe drugs they could follow throughout adult life that could reduce their late-life risk for AD a great deal further. The thesis of this essay is that a nutraceutical regimen comprised of agents that provide comprehensive antioxidant protection, support or mimic the bioactivity of NO, promote H_2_S biosynthesis, and aid neuroprotective M2 microglial differentiation, may have considerable potential in this regard. Moreover, such a regimen could be expected to have a favorable impact on cardiovascular health and on risk for diabetes and its complications, and hence would fit readily into a practical lifelong program for health promotion [63,308,309,310,311]. While it would be highly unrealistic to expect most people to cobble together such a regimen themselves, the incorporation of these nutraceuticals into functional foods and complex supplements might make such supplementation feasible.

## 5. Conclusions

In light of the wide range of cellular targets with which amyloid β can interact, and the mind-bending complexity of the pathogenesis of AD, defining optimal nutraceutical regimens for AD prevention represents a daunting task that will require considerable future investigation. Nonetheless, it is hoped that the strategies suggested here may represent a worthwhile start. Table 1 provides a list of the nutraceuticals with potential for AD prevention discussed herein, with dose ranges of each that seem likely to have clinical utility based on previous research.

## Figures and Tables

**Table 1:** Nutraceuticals with potential for prevention/control of Alzheimer’s Disease.

Nutraceuticals	Function	Typical Supplemental Dose/Day
Phycocyanobilin	Algal chromophore antioxidant	5–15 g spirulina
Lipoic Acid	Metabolic cofactor, phase two inducer	600 mg X 2–3
Melatonin	Neurohormone, phase two inducer	3–20 mg at bedtime
Taurine	Antioxidant/osmoregulatory cofactor	1–2 g X 2
N-Acetylcysteine	Supplemental source of L-cysteine	600 mg X 2–3
Selenium	Essential mineral	50–100 mcg
Astaxanthin	Natural carotenoid antioxidant	12–20 mg
Biotin	B vitamin, activator of guanylate cyclase	10–30 mg
Magnesium	Essential mineral	100–400 mg
Citrulline	Precursor/delivery form for arginine	2 g X 2
DHA	Long-chain omega-3 fatty acid	100–1000 mg
Vitamin D	Vitamin with anti-inflammatory activity	1000–5000 IU
Berberine	Phytochemical which activates AMPK	500 mg X 2–3

## Data Availability

Not applicable.

## Abbreviations

AD	Alzheimer’s disease
NAC	N-acetylcysteine
RYR2	ryanodine receptors
ER	endoplasmic reticulum
IRS-1	insulin receptor substrate-1
GLT-1	glutamate
PCB	Phycocyanobilin
MLT	melatonin
HO-1	heme oxygenase-1
H2S	hydrogen sulfide
CBS	cystathionine beta-synthase
Se	selenium
AST	astaxanthin
ETC	electron transport chain
APP	amyloid precursor protein
sGC	soluble guanylate cyclase
NO	nitric oxide
PPARα	peroxisome proliferator-activated receptor α
PKC	protein kinase C
IDE	insulin-degrading enzyme
P-GP	P-glycoprotein
eNOS	endothelial nitric oxide synthase
DDAH	dimethylarginine dimethylaminohydrolase
ADMA	asymmetric dimethylarginine
IL-1β	interleukin-1β
Mg	Magnesium
DHA	docosahexaenoic acid
NMDA	N-methyl-D-aspartate
JNK	c-Jun N-terminal kinase
ASK1	apoptosis signal-regulating kinase 1
NADPH	Reduced form of Nicotinamide adenine dinucleotide phosphate
LRP1	lipoprotein receptor-related protein 1
GFAP	glial fibrillary acidic protein
EGCG	epigallocatechin-3-gallate
SREBP-2	sterol regulatory element-binding protein 2
RAGE	receptor for advanced glycation end products
LTP	Long-term potentiation
TLR	Toll-like receptor
HMGB1	high-mobility group box protein 1
FGF21	fibroblast growth factor 21
LA	Lipoic acid
